# Job displacement and subjective well-being: findings from the American Time Use Survey Well-Being Modules

**DOI:** 10.1186/s12651-018-0249-5

**Published:** 2018-11-30

**Authors:** Younghwan Song

**Affiliations:** 1 Department of Economics, Union College, Schenectady, NY 12308 USA; 2 IZA, Bonn, Germany

**Keywords:** Job displacement, Subjective well-being, Cantril ladder, Net affect, J63, J65, I31

## Abstract

Using matched cross-sectional data drawn from the 2010 and 2012 Displaced Workers Supplements of the Current Population Surveys and the 2010, 2012, and 2013 American Time Use Survey Well-Being Modules, this paper examines the relationship between job displacement and various measures of subjective well-being by sex. Displaced men report lower levels of life evaluation than nondisplaced men due to the differences in employment, marital status and income, whereas displaced women report lower levels of net affect and happiness and increased pain, sadness, and stress than nondisplaced women, but no difference in their life evaluation. Among men, those displaced by layoffs, not by plant closings, express lower levels of life evaluation than those not displaced, but there is no such difference by cause of displacement among women. The negative relationship between job displacement and subjective well-being decreases over time for both men and women.

## Introduction

Since the seminal paper by Clark and Oswald (1994), economists have been increasingly interested in the connection between unemployment and subjective well-being. In an effort to test the hypothesis that the rising unemployment in Britain is voluntary, Clark and Oswald (1994) analyze mental well-being scores from the British Household Panel Study. Finding that unemployed people have lower levels of mental well-being than those employed, they conclude that unemployment is not voluntary in general. As they acknowledge, however, it is difficult to establish causality with their cross-sectional data. For example, people with intrinsically lower levels of subjective well-being are more likely to lose jobs. To control for such unobserved individual heterogeneity, other researchers employ individual fixed effects on panel data from the German Socio-Economic Panel (GSOEP) and provide causal evidence that unemployment does significantly lower life satisfaction (Clark et al. 2001; Gerlach and Stephan 1996; Kassenboehmer and Haisken-DeNew 2009; Winkelmann and Winkelmann 1998). Kassenboehmer and Haisken-DeNew (2009) further point out that a decrease in job satisfaction may cause a worker to become voluntarily unemployed, rendering the fixed-effect estimates based on panel data still biased. To overcome this problem, they identify the workers in their data who are involuntarily unemployed due to company closures and show that this involuntary entry into unemployment further lowers life satisfaction among German women, but not among German men.^1^ Interestingly, the magnitudes of the effects of unemployment on life satisfaction are very similar between the fixed-effects estimations and those without fixed effects in most papers, suggesting that the bias due to unobserved individual heterogeneity is minimal in the pooled estimations without fixed effects. Due to the lack of data, however, just one cognitive measure of subjective well-being, overall satisfaction with life, is analyzed in the papers based on the GSOEP.

Knabe et al. (2010), using a cross-sectional survey of long-term unemployed from Germany, first analyze the relationship between unemployment and affective measures of subjective well-being based on time use, such as the U-index and net affect, in addition to life satisfaction. They find that unemployed people are less satisfied with their life and report more negative feelings when engaged in similar activities than those who are employed, but duration-weighted average day-to-day subjective well-being is not different between the two groups because the unemployed use that time during which the employed are at work in more enjoyable ways. In contrast, Krueger and Mueller (2012), based on weekly panel data of unemployed workers in New Jersey, find that the unemployed feel sadder over the course of the entire day than the employed and the estimates are similar regardless of controlling for unobserved individual heterogeneity by using fixed effects. They point out that the difference in findings between them and Knabe et al. (2010) might be because Knabe et al. (2010) do not include sadness in their affective measures in constructing the U-index and net affect. Nevertheless, neither paper differentiates voluntary and involuntary entry into unemployment.

It is well established that displaced workers—individuals who lost their jobs involuntarily due to plant closings or mass layoffs—incur significant economic costs: long-term earnings losses and job instability (Jacobson et al. 1993; Stevens 1997). A growing literature shows that involuntary job losers also experience additional non-economic consequences: higher rates of divorce (Charles and Stephens 2004; Doiron and Mendolia 2012; Eliason 2012); worse health in general, including self-rated health, mental health and depression (Black et al. 2015; Brand et al. 2008; Gallo et al. 2000; Bungard et al. 2007; Gallo et al. 2006a, b; Schaller and Stevens 2015; Strully 2009); and higher mortality rates, in particular from suicides (Browning and Heinesen 2012; Eliason and Storrie 2009; Keefe et al. 2002; Noelke and Beckfield 2014; Sullivan and Wachter 2009). Using linked data from the 2010 and 2012 Displaced Workers Supplements (DWS) to the Current Population Surveys (CPS) and the 2010, 2012, and 2013 American Time Use Survey Well-Being (ATUS WB) Modules, this paper contributes to the literature by providing first empirical evidence on the relationship between job displacement and various measures of subjective well-being.

In addition to the often-analyzed standard life evaluation question using the Cantril ladder, this paper also examines various measures of subjective well-being based on time use—the U-index, net affect, meaningfulness, happiness, pain, sadness, and stress—and self-rated health status. The U-index, net affect, happiness, pain, sadness, and stress all measure the presence of pleasure and the absence of displeasure, which corresponds to affective (or hedonistic) views of subjective well-being, while meaningfulness and the Cantril ladder measure a cognitive state or a positive attitude towards one’s life, which corresponds to cognitive (or attitudinal) views of subjective well-being (Angner 2010; Brülde 2007). By analyzing these various measures of subjective well-being, this paper can help provide a fuller picture of the link between job displacement and subjective well-being.

The existing literature on job displacement also documents that the effect of job displacement varies by cause of displacement and by gender. For example, Gibbons and Katz (1991), based on data from the 1984 and 1986 DWS, show that even though the predisplacement wages do not differ by cause of displacement, male workers displaced by layoffs receive lower post-displacement wages, incur larger earnings losses, and have longer unemployment spells than those displaced by plant closings. According to Gibbons and Katz, it is because when firms have discretion with respect to whom to lay off, a layoff event signals that laid-off workers are of low ability.^2^ In the same context, Charles and Stephens (2004) show that the increase in divorce is found only for men who were laid off, within 3 years of job displacement, but not for men displaced by plant closings. Finally, Brand et al. (2008) find that among older workers, men have significant increases in depression as a result of layoffs, but not as a result of plant closings, while the reverse is the case among women. So, this paper also examines how the relationship between displacement and subjective well-being varies by cause of displacement and by gender. This separate analysis by gender also allows revisiting the findings in psychology literature that women tend to show more positive and negative emotions than men (Chaplin and Aldao 2013; Fujita et al. 1991; Mestre et al. 2009) as well as the different effects by gender of company closures on life satisfaction reported in Kassenboehmer and Haisken-DeNew (2009).

Because the ATUS WB Modules used in this paper are cross-sectional, the estimates reported in this paper might be, rather than showing unbiased causal effects, subject to unobserved individual heterogeneity, as pointed out above. The existing literature shows that unhappy workers are less productive (Oswald et al. 2015; Wright and Cropanzano 2000) and less productive workers are more likely to be laid off (Gibbons and Katz 1991). In order to sort out whether job displacement is really causing lower levels of subjective well-being, ideally it is necessary to employ individual fixed effects using panel data that spans the periods before and after displacement. Because panel data is not available, this paper employs additional analyses using the number of years between job displacement and the ATUS WB Module interview, which provided supportive evidence for causal interpretation of the results. Furthermore, given the consistent findings in the literature that the magnitudes of the effects of unemployment on diverse measures of subjective well-being are very similar between the fixed-effects estimations and those without fixed effects (Clark et al. 2001; Gerlach and Stephen 1996; Kassenboehmer and Haisken-DeNew 2009; Krueger and Mueller 2012; Winkelmann and Winkelmann 1998), the cross-sectional estimates in this paper, although not guaranteed, might not suffer too much from endogeneity.^3^ If that is the case, the estimates in this paper are likely to be rather conservative. First, because there could be a gap between 2 to 20 months between the DWS and the ATUS WB Modules, it is possible that some of the nondisplaced workers in the DWS could have experienced displacement by the time they interviewed for the ATUS WB Modules but are still categorized as the nondisplaced in the analysis. As a result, the differences in subjective well-being between the displaced and the nondisplaced are likely to be underestimated. Second, hedonic adaptation points out that people tend to go back to a new level of indifference after transient changes in subjective well-being due to major positive or negative events in life (Brickman and Campbell 1971). It is possible that after the initial shock from displacement, displaced workers might have returned to a relatively stable level of subjective well-being over time through hedonic adaptation. Then, the estimated effects of job displacement in this paper are likely to have a downward bias.

The remainder of the paper is organized as follows. In Sect. 2, I describe the data and the econometric model. Section 3 presents the empirical results and discusses the findings. Finally, Sect. 4 provides concluding remarks.

## Data and methodology

This study uses cross-sectional matched data drawn from the DWS to the 2010 and 2012 January CPS and the 2010, 2012, and 2013 ATUS WB Modules. Details of the matching of the sample of displaced and nondisplaced respondents from the DWS and the ATUS WB Modules are reported in Appendix. The DWS has been conducted biennially since 1984 and is a key source of national level data on worker displacement in the United States. The 2010 and 2012 DWS asked individuals at least 20 years of age if they have lost or left a job in the prior 3 years because of a plant closing, slack work, a position or shift abolished, or other reasons.^4^ From the workers who answered affirmatively, detailed information was collected about the predisplacement job and the year of displacement. Following the literature on worker displacement, those displaced because of slack work or a position or shift abolished are classified as layoffs (Gibbons and Katz 1991). Following Farber (1993, 1997, 2011), I have also treated nondisplaced workers employed as of the survey date from the 2010 and 2012 DWS as the reference group—the relevant pool of workers who were at risk of losing a job during the time-period of the prior 3 years. The displaced workers from the 2010 and 2012 DWS involuntarily lost their jobs over the years from 2007 through 2011, when job loss rates increased drastically during the Great Recession (Farber 2011).

The ATUS WB Module is a module added to the American Time Use Survey (ATUS), a time-diary study conducted continuously since 2003 by the U.S. Census Bureau, based on a nationally representative cross-sectional sample of the U.S. population 15 years of age or older. Through telephone interviewing, the ATUS collects a detailed account of respondents’ activities on a 24-h, preassigned day of the week (the diary day). The diary days of the ATUS include all days in a year, except Thanksgiving Day and Christmas Day.

In the 2010, 2012 and 2013 ATUS WB Modules, the survey randomly selected three activities reported by each respondent of the ATUS^5^ and for each selected activity asked five affect questions (happiness, pain, sadness, stress, and tiredness) and one question about how meaningful the activity was. For each of these questions, respondents were asked to respond using a scale from 0 to 6, where a 0 would mean he/she did not experience the feeling at all and a 6 would mean the feeling was very strong. In addition, the 2012 and 2013 ATUS WB Modules also have a standard life-evaluation question using the Cantril ladder (Cantril 1965). It first asked respondents to imagine a ladder where ten steps increasingly numbered from 0 to 10 to the top, with the bottom of the ladder representing the worst possible life for them while the top of the ladder representing the best possible life for them. After being given this, respondents were asked on which step of the ladder they personally feel they stand at this time. The 2010, 2012 and 2013 ATUS WB Modules also contain four general health questions: self-rated general health status (excellent, very good, good, fair, and poor); whether the respondent was ever diagnosed with hypertension by a doctor in the last 5 years; whether the respondent took any pain medication on the diary day; and how well rested the respondent felt on the diary day.

Using the responses available in the 2010, 2012 and 2013 ATUS WB Modules, I have constructed two composite measures of individual-level subjective well-being: the U-index and net affect. Following Kahneman and Krueger (2006) and Krueger et al. (2009), I classified an episode as unpleasant if the highest rating on any of the three negative affect dimensions (pain, sadness, and stress) is strictly greater than the rating of the positive affect dimension (happiness). Then the U-index for a respondent *i*, *U*_*i*_, is constructed as the weighted average of these classifications over the episodes from the respondent as follows

1 $$U_{i} = \frac{{\mathop \sum \nolimits_{k} w_{ik} U_{ik} }}{{\mathop \sum \nolimits_{k} w_{ik} }}$$

where *i* denotes the respondent, *k* denotes the sampled activity, *U*_*ik*_ denotes an indicator variable for an episode *k* being unpleasant for the respondent *i*, and *w*_*ik*_ denotes the WB Module adjusted pooled activity weight (WUFNACTWTCP) attached to activity *k* for respondent *i*. The WB Module adjusted pooled activity weights account for both (i) differences between activities in the fraction of time spent in eligible activities and (ii) differences between persons in the probability of having a specific eligible activity selected due to variation in the number of eligible activities. This U-index is an estimate for the fraction of time the respondent spends in an unpleasant state.

I define net affect for each episode as the difference between the positive emotion (happiness) and the average of the negative ones (pain, sadness, and stress) for the episode (Kahneman et al. 2004). Using a formula similar to Eq. 1, I defined net affect for each individual as the weighted average of net affect over the activities from the respondent. I have also constructed meaningfulness for each respondent as the weighted average of the responses to the question about how meaningful the episode was over the episodes from the respondent. In the same way, I have constructed happiness, pain, sadness, and stress for each respondent. And to compare the health outcomes, I also analyze self-rated general health status. For a subsample from the 2012 and 2013 ATUS WB Modules, I use the responses to the Cantril ladder as another measure of subjective well-being. After excluding the episodes with missing responses, there are 6051 respondents (2942 men and 3109 women) who are 20 years of age or older in the final sample and 560 of them (301 men and 259 women), 9.25 percent, are displaced workers.^6^

Using each of the measures of subjective well-being as the dependent variable, I first estimated Ordinary Least Squares (OLS) regressions by gender. In the baseline model, I control for the following respondents’ characteristics: age and its square; three dummies for race/ethnicity; five education dummies; number of disabilities^7^; an immigrant dummy; number of children; a metropolitan status dummy; two season dummies; a holiday dummy; six dummies for the days of the week; year dummies; dummies for the DWS year; and state dummies. When the responses to the Cantril ladder or self-rated health status are used as the dependent variable, I exclude a holiday dummy, and six dummies for the days of the week.^8^ Then, in another set of analyses, I additionally included independent variables that are likely to have been affected by the incident of job displacement: two marital-status dummies; two employment-status dummies; and four dummies for family income during the last 12 months as of the last CPS interview date^9^ (Charles and Stephens 2004; Doiron and Mendolia 2012; Eliason 2012; Jacobson et al. 1993; Stevens 1997). These additional analyses would sort out whether the link between job displacement and subjective well-being is only through the changes of employment status, marital status, and income or through other mechanisms beyond the changes in employment, marital status and income.

## Results

Table 1 reports descriptive statistics by sex and displacement status, weighted using the ATUS WB Module final weight (WUFINLWGT). Among men, those displaced have significantly lower levels of the Cantril ladder than those nondisplaced, whereas there is no significant difference in other measures of subjective well-being. In contrast, among women, those displaced have significantly lower levels of net affect than those nondisplaced because the displaced are less happy but feel more pain and sadness than the nondisplaced, while there is no significant difference in the Cantril ladder between the two groups.

**Table 1:** Descriptive statistics by sex and displacement status

	Men		Women	
	Displaced	Nondisplaced	Displaced	Nondisplaced
Dependent variables				
U-index	0.180 (0.025)	0.150 (0.008)	0.197 (0.029)	0.150 (0.007)
Net affect	3.244 (0.147)	3.337 (0.050)	2.929 (0.190)	3.446 (0.050)***
Meaningfulness	4.199 (0.144)	4.282 (0.039)	4.273 (0.151)	4.370 (0.040)
Happiness	4.183 (0.099)	4.229 (0.036)	4.107 (0.144)	4.367 (0.034)*
Pain	0.877 (0.119)	0.735 (0.031)	1.059 (0.128)	0.764 (0.034)**
Sadness	0.607 (0.086)	0.518 (0.027)	0.829 (0.131)	0.513 (0.027)**
Stress	1.331 (0.107)	1.420 (0.039)	1.648 (0.141)	1.487 (0.041)
Cantril ladder	6.493 (0.189)	7.066 (0.060)***	7.216 (0.186)	7.316 (0.058)
Self-rated health	3.537 (0.073)	3.664 (0.024)*	3.601 (0.092)	3.637 (0.024)
Independent variables				
Layoff	0.696 (0.034)		0.726 (0.036)	
Plant closing	0.304 (0.034)		0.274 (0.036)	
Age	44.584 (0.900)	44.005 (0.381)	44.089 (1.046)	44.034 (0.364)
White	0.666 (0.033)	0.721 (0.012)	0.670 (0.038)	0.713 (0.011)
Black	0.094 (0.018)	0.079 (0.006)	0.128 (0.024)	0.111 (0.007)
Hispanic	0.159 (0.024)	0.143 (0.009)	0.130 (0.026)	0.110 (0.008)
Other	0.080 (0.021)	0.057 (0.008)	0.072 (0.023)	0.065 (0.007)
Elementary school	0.040 (0.013)	0.031 (0.005)	0.030 (0.015)	0.016 (0.003)
Some high school	0.109 (0.029)	0.053 (0.006)*	0.051 (0.017)	0.027 (0.004)
High school	0.326 (0.035)	0.297 (0.012)	0.207 (0.034)	0.259 (0.012)
Some college	0.216 (0.028)	0.256 (0.011)	0.398 (0.040)	0.286 (0.011)***
College	0.236 (0.031)	0.242 (0.011)	0.231 (0.036)	0.250 (0.011)
Graduate	0.073 (0.016)	0.122 (0.007)***	0.083 (0.022)	0.162 (0.009)***
Number of disabilities	0.089 (0.034)	0.050 (0.007)	0.097 (0.029)	0.044 (0.006)*
Number of children	0.693 (0.072)	0.692 (0.025)	0.597 (0.070)	0.644 (0.023)
Immigrant	0.172 (0.026)	0.153 (0.010)	0.163 (0.030)	0.122 (0.009)
Metropolitan status	0.838 (0.026)	0.827 (0.010)	0.879 (0.030)	0.835 (0.009)
Single	0.336 (0.035)	0.296 (0.012)	0.365 (0.037)	0.355 (0.012)
Married	0.607 (0.036)	0.651 (0.013)	0.524 (0.041)	0.582 (0.012)
Partnered	0.057 (0.018)	0.053 (0.006)	0.111 (0.032)	0.063 (0.007)
Employed	0.615 (0.037)	0.947 (0.006)***	0.668 (0.037)	0.929 (0.006)***
Unemployed	0.257 (0.035)	0.020 (0.004)***	0.141 (0.026)	0.022 (0.004)***
Not in labor force	0.128 (0.024)	0.033 (0.005)***	0.190 (0.030)	0.048 (0.005)***
Family income: less than $30,000	0.304 (0.035)	0.151 (0.009)***	0.389 (0.039)	0.169 (0.009)***
$30,000–60,000	0.287 (0.032)	0.281 (0.011)	0.281 (0.038)	0.286 (0.012)
$60,000–100,000	0.254 (0.032)	0.297 (0.012)	0.182 (0.032)	0.299 (0.012)***
$100,000–150,000	0.070 (0.018)	0.161 (0.010)***	0.081 (0.024)	0.153 (0.009)***
More than $150,000	0.086 (0.019)	0.109 (0.008)	0.068 (0.028)	0.094 (0.007)
Observations	301	2641	259	2850

The numbers of observations for the Cantril ladder are 178 displaced workers and 1591 nondisplaced workers among men and 148 displaced workers and 1698 nondisplaced workers among women. All statistics use the WB Module final weight (WUFINLWGT). Standard errors are in parentheses

*, **, *** Denote the means are significantly different between the displaced and the nondisplaced at the 10, 5, and 1% level, respectively

Table 1 also shows that more than two thirds of the displaced workers lost their jobs due to layoffs, regardless of sex. Except that the nondisplaced are slightly better educated than the displaced, regardless of sex, the displaced and the nondisplaced are similar in other characteristics, such as age, race/ethnicity, number of children, and immigrant status. Corroborating with the findings in Charles and Stephens (2004), Doiron and Mendolia (2012), and Eliason (2012), the displaced are less likely to be married than the nondisplaced, regardless of sex, though the difference is not statistically significant. Furthermore, as consistently found in the literature on displaced workers (Jacobson et al. 1993; Stevens 1997), displaced workers are significantly less likely to be employed and more likely to be unemployed or not in the labor force than the nondisplaced, regardless of sex. Finally, the nondisplaced tend to have larger family incomes than the displaced, regardless of sex.

Table 2 reports the coefficients on the job displacement dummy of OLS estimation for men. In Panel A, the results for the baseline model are reported, Panel B reports the results for the baseline model with two marital-status dummies; two employment-status dummies; and four dummies for family income. The estimation results in Panel A of Table 2 indicate that there is no significant difference between displaced and nondisplaced men in all measures of subjective well-being, except that in column 8 the levels of the Cantril ladder are significantly lower for the displaced than for the nondisplaced. In contrast, when marital status, employment status and family income are additionally controlled for in Panel B of Table 2, there is no significant difference even in the Cantril ladder by displacement status, suggesting that the differences in these additional characteristics between the displaced and the nondisplaced explain the lower life evaluation for displaced workers observed in Panel A of Table 2. Specifically, the conditional decomposition method by Gelbach (2016) shows that of the change of 0.246 between Panels A and B on the coefficient on displacement in column 8, marital status accounts for 0.023, employment status accounts for 0.169, and family income accounts for 0.053. Therefore, the differences in employment status seem to be the main reason for the lower level of the Cantril ladder for the unemployed.

**Table 2:** Coefficients on job displacement in the subjective well-being regressions: men

Variables	(1)	(2)	(3)	(4)	(5)	(6)	(7)	(8)	(9)
	U-index	Net affect	Meaningfulness	Happiness	Pain	Sadness	Stress	Cantril ladder	Self-rated health
*Panel A baseline model*									
Displacement	0.025 (0.025)	− 0.059 (0.148)	−0.048 (0.128)	−0.045 (0.101)	0.096 (0.118)	0.013 (0.086)	− 0.066 (0.107)	− 0.476** (0.190)	−0.034 (0.072)
Observations	2942	2942	2942	2942	2942	2942	2942	1769	2942
R-squared	0.061	0.075	0.072	0.081	0.096	0.080	0.085	0.114	0.106
*Panel B baseline model plus dummies for marital status, employment status, and income*									
Displacement	0.018 (0.026)	− 0.026 (0.152)	− 0.068 (0.120)	− 0.064 (0.107)	0.048 (0.114)	− 0.014 (0.092)	− 0.14 9 (0.111)	− 0.230 (0.179)	0.003 (0.071)
Observations	2942	2942	2942	2942	2942	2942	2942	1769	2942
R-squared	0.066	0.084	0.079	0.092	0.102	0.087	0.088	0.159	0.120

The estimates in columns 1 through 7 and 9 are OLS coefficients from the 2010, 2012, and 2013 ATUS WB Modules; the estimates in column 8 are OLS coefficients from the 2012 and 2013 ATUS WB Modules. All estimates use the WB Module final weight (WUFINLWGT). Standard errors in parentheses. *** p < 0.01, ** p < 0.05, * p < 0.1. The following control variables are included in all columns in both Panels A and B: age and its square; three dummies for race/ethnicity; five education dummies; number of disabilities; an immigrant dummy; number of children; a metropolitan status dummy; two season dummies; year dummies; dummies for the DWS year; and state dummies. Columns 1 through 7 additionally contain a holiday dummy and six dummies for the days of the week. Panel B additionally includes two marital-status dummies; two employment-status dummies; and four dummies for family income during the last 12 months

Table 3 shows the results for women. The baseline model in Panel A shows that displaced women have significantly lower levels of net affect than nondisplaced women, resulting from lower levels of happiness and higher levels of pain and sadness of the displaced than the nondisplaced, which is similar to the findings in Krueger and Mueller (2012) that the unemployed feel sadder than the employed. In contrast to the results for men in Table 2, the significant difference in net affect between displaced and nondisplaced women still remains even after controlling for employment status, marital status, and family income in Panel B of Table 3. Furthermore, unlike the results for men in Table 2, there is no difference in the Cantril ladder by displacement status among women in column 8 of Table 3, regardless of the inclusion of the additional control variables.

**Table 3:** Coefficients on job displacement in the subjective well-being regressions: women

Variables	(1)	(2)	(3)	(4)	(5)	(6)	(7)	(8)	(9)
	U-index	Net affect	Meaningfulness	Happiness	Pain	Sadness	Stress	Cantril ladder	Self-rated health
*Panel A baseline model*									
Displacement	0.043 (0.028)	− 0.491*** (0.185)	− 0.113 (0.137)	− 0.247* (0.140)	0.257** (0.125)	0.284** (0.123)	0.191 (0.140)	0.031 (0.191)	0.033 (0.083)
Observations	3109	3109	3109	3109	3109	3109	3109	1846	3109
R-squared	0.060	0.086	0.118	0.092	0.075	0.060	0.087	0.070	0.137
*Panel B baseline model plus dummies for marital status, employment status, and income*									
Displacement	0.056* (0.030)	− 0.525*** (0.192)	− 0.112 (0.145)	− 0.260* (0.147)	0.246** (0.124)	0.259** (0.125)	0.288** (0.141)	0.084 (0.198)	0.103 (0.077)
Observations	3109	3109	3109	3109	3109	3109	3109	1846	3109
R-squared	0.063	0.094	0.124	0.099	0.081	0.069	0.099	0.099	0.155

The estimates in columns 1 through 7 and 9 are OLS coefficients from the 2010, 2012, and 2013 ATUS WB Modules; the estimates in column 8 are OLS coefficients from the 2012 and 2013 ATUS WB Modules. All estimates use the WB Module final weight (WUFINLWGT). Standard errors in parentheses. *** p < 0.01, ** p < 0.05, * p < 0.1. The following control variables are included in all columns in both Panels A and B: age and its square; three dummies for race/ethnicity; five education dummies; number of disabilities; an immigrant dummy; number of children; a metropolitan status dummy; two season dummies; year dummies; dummies for the DWS year; and state dummies. Columns 1 through 7 additionally contain a holiday dummy and six dummies for the days of the week. Panel B additionally includes two marital-status dummies; two employment-status dummies; and four dummies for family income during the last 12 months

Overall, these results in Tables 2 and 3 indicate that job displacement is associated with lower levels of cognitive (or attitudinal) measures of subjective well-being, such as the Cantril ladder (Angner 2010; Brülde 2007), among men through the differences in employment, marriage, and income, whereas it is associated with only affective (or hedonistic) measures of subjective well-being among women beyond the differences in employment, marriage, and income.^10^ These findings are also consistent with the findings that women show more positive and negative emotions than men (Chaplin and Aldao 2013; Fujita et al. 1991; Mestre et al. 2009).

Contrary to the findings in Bungard et al. (2007) and Schaller and Stevens (2015), there is no evidence in column 9 of Tables 2 and 3 that job displacement is associated with poorer self-reported health, regardless of sex. The difference may stem from that this paper examines short-term—at most 4 years—effects of job displacement, whereas both Bungard et al. (2007) and Schaller and Stevens (2015) examine long-term—15 to 28 years—effects of job displacement on health.

To examine if the relationship between job displacement and various measures of subjective well-being observed in Tables 2 and 3 vary by cause of displacement, Tables 4 and 5 report the estimation results for men and women, respectively, where dummies for layoffs and plant closings are included, instead of the displacement dummy. The results for men in column 8 of Panel A in Table 4 indicate that those displaced by layoffs have significantly lower levels of the Cantril ladder than those not displaced but the difference is not significant between those displaced by plant closings and those not displaced. In column 8 of Panel B in Table 4, the coefficient on layoffs becomes insignificant when marital status, employment status, and family income are additionally controlled for, with employment status accounting for about 0.141 of the change of 0.234 in the coefficient on layoffs between Panels A and B in Table 4, based on the conditional decomposition method by Gelbach (2016). These results in Table 4 corroborate the findings in Gibbons and Katz (1991) that male workers displaced by layoffs incur larger earnings losses and have longer unemployment spells than those displaced by plant closings.

**Table 4:** Coefficients on Layoffs and Plant closings in the subjective well-being regressions: men

Variables	(1)	(2)	(3)	(4)	(5)	(6)	(7)	(8)	(9)
	U-index	Net affect	Meaningfulness	Happiness	Pain	Sadness	Stress	Cantril ladder	Self-rated health
*Panel A baseline model*									
Layoff	0.032 (0.028)	− 0.149 (0.165)	− 0.253 (0.157)	− 0.075 (0.113)	0.203 (0.128)	0.058 (0.097)	− 0.039 (0.130)	− 0.541** (0.213)	− 0.026 (0.075)
Plant closing	0.009 (0.047)	0.151 (0.287)	0.431** (0.177)	0.026 (0.188)	− 0.153 (0.243)	− 0.092 (0.166)	− 0.129 (0.173)	− 0.336 (0.360)	− 0.054 (0.149)
Observations	2942	2942	2942	2942	2942	2942	2942	1769	2942
R-squared	0.061	0.075	0.076	0.081	0.097	0.081	0.085	0.115	0.106
*Panel B baseline model plus dummies for marital status, employment status, and income*									
Layoff	0.023 (0.030)	− 0.095 (0.172)	− 0.250* (0.146)	− 0.076 (0.119)	0.149 (0.134)	0.025 (0.100)	− 0.119 (0.132)	− 0.306 (0.196)	0.005 (0.077)
Plant closing	0.004 (0.045)	0.144 (0.278)	0.381** (0.185)	− 0.035 (0.191)	− 0.202 (0.214)	− 0.111 (0.171)	− 0.224 (0.175)	− 0.052 (0.343)	− 0.001 (0.138)
Observations	2942	2942	2942	2942	2942	2942	2942	1769	2942
R-squared	0.066	0.084	0.083	0.092	0.104	0.087	0.088	0.159	0.120

The estimates in columns 1 through 7 and 9 are OLS coefficients from the 2010, 2012, and 2013 ATUS WB Modules; the estimates in column 8 are OLS coefficients from the 2012 and 2013 ATUS WB Modules. All estimates use the WB Module final weight (WUFINLWGT). Standard errors in parentheses. *** p < 0.01, ** p < 0.05, * p < 0.1. The following control variables are included in all columns in both Panels A and B: age and its square; three dummies for race/ethnicity; five education dummies; number of disabilities; an immigrant dummy; number of children; a metropolitan status dummy; two season dummies; year dummies; dummies for the DWS year; and state dummies. Columns 1 through 7 additionally contain a holiday dummy and six dummies for the days of the week. Panel B additionally includes two marital-status dummies; two employment-status dummies; and four dummies for family income during the last 12 months

**Table 5:** Coefficients on Layoffs and Plant closings in the subjective well-being regressions: women

Variables	(1)	(2)	(3)	(4)	(5)	(6)	(7)	(8)	(9)
	U-index	Net affect	Meaningfulness	Happiness	Pain	Sadness	Stress	Cantril ladder	Self-rated health
*Panel A baseline model*									
Layoff	0.036 (0.034)	− 0.481** (0.228)	− 0.118 (0.173)	− 0.276 (0.179)	0.180 (0.145)	0.285** (0.143)	0.149 (0.163)	0.134 (0.226)	0.025 (0.099)
Plant closing	0.061 (0.051)	− 0.520* (0.285)	− 0.100 (0.196)	− 0.173 (0.169)	0.459** (0.234)	0.283 (0.234)	0.300 (0.256)	− 0.178 (0.335)	0.053 (0.139)
Observations	3109	3109	3109	3109	3109	3109	3109	1846	3109
R-squared	0.060	0.086	0.118	0.093	0.075	0.060	0.087	0.070	0.137
*Panel B baseline model plus dummies for marital status, employment status, and income*									
Layoff	0.049 (0.035)	− 0.519** (0.235)	− 0.117 (0.180)	− 0.292 (0.185)	0.179 (0.141)	0.261* (0.142)	0.240 (0.163)	0.155 (0.240)	0.085 (0.090)
Plant closing	0.073 (0.052)	− 0.540* (0.287)	− 0.098 (0.195)	− 0.178 (0.170)	0.419* (0.235)	0.255 (0.243)	0.411 (0.254)	− 0.063 (0.329)	0.149 (0.134)
Observations	3109	3109	3109	3109	3109	3109	3109	1846	3109
R-squared	0.063	0.094	0.124	0.100	0.081	0.069	0.099	0.099	0.155

The estimates in columns 1 through 7 and 9 are OLS coefficients from the 2010, 2012, and 2013 ATUS WB Modules; the estimates in column 8 are OLS coefficients from the 2012 and 2013 ATUS WB Modules. All estimates use the WB Module final weight (WUFINLWGT). Standard errors in parentheses. *** p < 0.01, ** p < 0.05, * p < 0.1. The following control variables are included in all columns in both Panels A and B: age and its square; three dummies for race/ethnicity; five education dummies; number of disabilities; an immigrant dummy; number of children; a metropolitan status dummy; two season dummies; year dummies; dummies for the DWS year; and state dummies. Columns 1 through 7 additionally contain a holiday dummy and six dummies for the days of the week. Panel B additionally includes two marital-status dummies; two employment-status dummies; and four dummies for family income during the last 12 months

Unexpectedly, those men displaced by plant closings in fact express higher levels of meaningfulness than those not displaced in column 3 of both Panels A and B in Table 4, regardless of the inclusion of the additional controls.

The results for women in column 2 of Panels A and B in Table 5 show that the magnitudes of the negative coefficients on net affect are similar between layoffs and plant closings, regardless of controlling for the additional independent variables in Panel B. These results for women are different not only from the findings based on men, such as Gibbons and Katz (1991) and Charles and Stephens (2004), but also from the varying effects of layoffs and plant closings on depression by sex—higher levels of depression among men as a result of layoffs, but not as a result of plant closings, whereas higher levels of depression among women as a result of plant closings, but not as a result of layoffs—found by Brand et al. (2008). Furthermore, the lack of significance on the coefficient on plant closing in column 8 of Panels A and B in Table 5 is different from the findings in Kassenboehmer and Haisken-DeNew (2009) that involuntary entry into unemployment due to company closures further lowers life satisfaction among German women.

Given the cross-sectional data used in this paper, one cannot argue that the estimates reported above are not due to unobserved individual heterogeneity, such as constantly lower levels of subjective well-being for some workers who, as a result, were laid off. Because no panel data are available here, however, this paper takes a different approach. In the DWS, displaced workers reported the year of their job loss. Using this information, I have created three dummies—1–2 years, 3–4 years, and missing^11^—for the number of years between the year of job displacement and the year of ATUS WB Module interview, in lieu of the displacement dummy. If displaced workers already had time-invariant, lower levels of subjective well-being than nondisplaced workers prior to their job displacement and as a result lost their jobs, the association between job loss and measures of subjective well-being would not change as time passes after displacement. However, if the lower levels of subjective well-being among the displaced are in fact due to job displacement, as more years pass after the event of displacement the effect of job displacement on subjective well-being would decrease because of hedonic adaptation (Brickman and Campbell 1971).

To examine if the negative effect of displacement on subjective well-being decreases as time passes, Tables 6 and 7 report the estimation results for men and women, respectively. The results for men in column 8 of Panel A of Table 6 show that the negative effect of displacement on the Cantril ladder weakens over time, while this pattern disappears in column 8 of Panel B of Table 6 when the additional controls for marital status, employment status, and income are included. The results for women in Panels A and B of Table 7 indicate that in the first 2 years after job displacement, women report significantly lower levels of net affect and higher levels of pain, sadness, and stress than nondisplaced women and the differences decrease over time, which is consistent with hedonic adaptation (Brickman and Campbell 1971). Although the missing dummies in Tables 6 and 7 show significant results for both men and women, it is difficult to make inference about them regarding hedonic adaptation.

**Table 6:** Coefficients on years since displacement in the subjective well-being regressions: men

Variables	(1)	(2)	(3)	(4)	(5)	(6)	(7)	(8)	(9)
	U-index	Net affect	Meaningfulness	Happiness	Pain	Sadness	Stress	Cantril ladder	Self− rated health
*Panel A baseline model*									
1–2 years	0.002 (0.031)	0.114 (0.190)	− 0.109 (0.145)	− 0.061 (0.133)	− 0.160 (0.129)	− 0.140 (0.091)	− 0.223* (0.124)	− 0.556** (0.261)	0.001 (0.079)
3–4 years	0.069* (0.041)	− 0.341 (0.228)	0.122 (0.250)	− 0.020 (0.146)	0.492** (0.223)	0.259 (0.166)	0.213 (0.180)	− 0.316 (0.261)	− 0.064 (0.131)
Missing	− 0.158* (0.081)	− 0.152 (0.704)	− 2.225*** (0.288)	− 0.010 (0.331)	0.939*** (0.318)	0.160 (0.509)	− 0.675 (0.889)	− 2.673*** (1.008)	− 0.902** (0.386)
Observations	2942	2942	2942	2942	2942	2942	2942	1769	2942
R-squared	0.063	0.076	0.075	0.081	0.102	0.083	0.086	0.117	0.107
*Panel B baseline model plus dummies for marital status, employment status, and income*									
1–2 years	− 0.007 (0.032)	0.138 (0.193)	− 0.161 (0.158)	− 0.093 (0.140)	− 0.209 (0.133)	− 0.162 (0.102)	− 0.322** (0.132)	− 0.276 (0.253)	0.022 (0.085)
3–4 years	0.060 (0.040)	− 0.282 (0.229)	0.112 (0.236)	− 0.023 (0.145)	0.432** (0.217)	0.213 (0.166)	0.133 (0.174)	− 0.156 (0.248)	− 0.014 (0.123)
Missing	− 0.205** (0.094)	0.127 (0.760)	− 2.269*** (0.379)	0.079 (0.382)	0.802** (0.349)	0.077 (0.532)	− 1.023 (0.919)	− 1.758* (1.040)	− 0.785** (0.385)
Observations	2942	2942	2942	2942	2942	2942	2942	1769	2942
R-squared	0.067	0.085	0.082	0.092	0.108	0.089	0.090	0.160	0.121

The estimates in columns 1 through 7 and 9 are OLS coefficients from the 2010, 2012, and 2013 ATUS WB Modules; the estimates in column 8 are OLS coefficients from the 2012 and 2013 ATUS WB Modules. All estimates use the WB Module final weight (WUFINLWGT). Standard errors in parentheses. *** p < 0.01, ** p < 0.05, * p < 0.1. The following control variables are included in all columns in both Panels A and B: age and its square; three dummies for race/ethnicity; five education dummies; number of disabilities; an immigrant dummy; number of children; a metropolitan status dummy; two season dummies; year dummies; dummies for the DWS year; and state dummies. Columns 1 through 7 additionally contain a holiday dummy and six dummies for the days of the week. Panel B additionally includes two marital-status dummies; two employment-status dummies; and four dummies for family income during the last 12 months

**Table 7:** Coefficients on years since displacement in the subjective well-being regressions: women

Variables	(1)	(2)	(3)	(4)	(5)	(6)	(7)	(8)	(9)
	U-index	Net affect	Meaningfulness	Happiness	Pain	Sadness	Stress	Cantril ladder	Self-rated health
*Panel A baseline model*									
1–2 years	0.058 (0.038)	− 0.673*** (0.233)	0.086 (0.140)	− 0.215 (0.166)	0.429** (0.174)	0.600*** (0.185)	0.345* (0.199)	− 0.226 (0.278)	0.012 (0.111)
3–4 years	0.008 (0.042)	− 0.102 (0.278)	− 0.271 (0.233)	− 0.204 (0.231)	− 0.048 (0.162)	− 0.166* (0.097)	− 0.092 (0.180)	0.370 (0.231)	0.085 (0.122)
Missing	0.252*** (0.087)	− 2.287*** (0.492)	− 2.686*** (0.929)	− 1.821*** (0.351)	0.886*** (0.315)	− 0.397*** (0.135)	0.910*** (0.254)	− 2.206*** (0.277)	− 0.275 (0.224)
Observations	3109	3109	3109	3109	3109	3109	3109	1846	3109
R-squared	0.061	0.089	0.124	0.095	0.077	0.068	0.089	0.075	0.137
*Panel B baseline model plus dummies for marital status, employment status, and income*									
1–2 years	0.073* (0.040)	− 0.706*** (0.243)	0.090 (0.142)	− 0.220 (0.173)	0.417** (0.172)	0.576*** (0.189)	0.464** (0.202)	− 0.138 (0.272)	0.102 (0.102)
3–4 years	0.017 (0.042)	− 0.137 (0.274)	− 0.253 (0.234)	− 0.219 (0.230)	− 0.051 (0.159)	− 0.178* (0.101)	− 0.018 (0.173)	0.424* (0.238)	0.129 (0.113)
Missing	0.270*** (0.088)	− 2.442*** (0.476)	− 2.723*** (0.948)	− 1.911*** (0.336)	0.916*** (0.313)	− 0.332** (0.153)	1.009*** (0.260)	− 2.530*** (0.278)	− 0.277 (0.219)
Observations	3109	3109	3109	3109	3109	3109	3109	1846	3109
R-squared	0.064	0.097	0.130	0.102	0.083	0.077	0.100	0.106	0.155

The estimates in columns 1 through 7 and 9 are OLS coefficients from the 2010, 2012, and 2013 ATUS WB Modules; the estimates in column 8 are OLS coefficients from the 2012 and 2013 ATUS WB Modules. All estimates use the WB Module final weight (WUFINLWGT). Standard errors in parentheses. *** p < 0.01, ** p < 0.05, * p < 0.1. The following control variables are included in all columns in both Panels A and B: age and its square; three dummies for race/ethnicity; five education dummies; number of disabilities; an immigrant dummy; number of children; a metropolitan status dummy; two season dummies; year dummies; dummies for the DWS year; and state dummies. Columns 1 through 7 additionally contain a holiday dummy and six dummies for the days of the week. Panel B additionally includes two marital-status dummies; two employment-status dummies; and four dummies for family income during the last 12 months

The results in Tables 6 and 7 suggest that the variation in subjective well-being by displacement status found in this paper is not likely due to individual heterogeneity or reverse causality. If those workers with constantly lower levels of subjective well-being were more likely to be displaced, their levels of subjective well-being are unlikely to have changed as time passes. Furthermore, the similarity of the effects of unemployment on diverse measures of subjective well-being between the panel fixed-effects estimations and those without fixed effects in the literature is reassuring (Clark et al. 2001; Gerlach and Stephen 1996; Kassenboehmer and Haisken-DeNew 2009; Krueger and Mueller 2012; Winkelmann and Winkelmann 1998). Overall, the cross-sectional estimates in this paper, although not guaranteed, seem to suggest causal effects of displacement on subjective well-being, without being biased too much due to endogeneity.

## Conclusions

In an effort to provide a fuller picture of the link between job displacement and subjective well-being, this paper has examined the relationship between job displacement and various measures of subjective well-being based on time-diary data—the U-index, net affect, meaningfulness, happiness, pain, sadness, and stress—as well as a standard life evaluation question using the Cantril ladder and self-rated health status. The results indicate that the relationship between job displacement and subjective well-being varies by sex and by measure of subjective well-being: among men involuntary job loss is not associated with moment-to-moment subjective well-being but is associated with lower levels of life evaluation through differences in employment, marital status and income, whereas among women job displacement is associated with lower levels of net affect, mostly due to decreased happiness and increased pain, sadness, and stress, but is not associated with life evaluation and, unlike men, the significant difference in net affect between displaced and nondisplaced women still exists even after controlling for employment status, marital status, and income. Among men, those displaced by layoffs, not by plant closings, express lower levels of the Cantril ladder than those not displaced but there is no such difference by cause of displacement among women. The negative associations between job displacement and subjective well-being decrease over time for both men and women, providing support for the argument that the association between job displacement and levels of subjective well-being is not all due to individual heterogeneity or reverse causality. Finally, no negative relationship between job displacement and self-rated health was found in this paper. This differs from the findings in the extant literature, perhaps as the period under consideration in this paper is relatively short.

In addition to the poor economic outcomes and marital dissolution among displaced workers found in the existing literature, the findings in this paper illustrate that the experience of job displacement also lowers subjective well-being. Displaced men have lower levels of life evaluation than those not displaced. But, after controlling for employment status, marital status, and income, there is no link between job displacement and any measure of subjective well-being among men. Among women, however, even after controlling for those variables, the negative relationship between involuntary job loss and affective measures of subjective well-being still remains, indicating that women incur additional psychological and emotional costs of job displacement, on top of the economic and marital costs.

One of the main limitations of this paper is that ATUS WB Modules used in this paper are cross-sectional. It is helpful that the additional analyses using the number of years between job displacement and the ATUS WB Module interview provided supportive evidence for causal interpretation. Also the similarity of the estimates using panel data with and without controlling for unobserved heterogeneity in the literature is also supportive of the causal interpretation of the estimates in this paper. However, it cannot be denied that one would ideally need panel data that spans the periods before and after displacement to get unbiased causal estimates.

Another limitation of this paper is that many of the displaced workers in the sample lost their jobs during the Great Recession, around the period between 2007 and 2009 when the unemployment rate peaked at ten percent in the United States. As a result, these displaced workers are certainly likely to have had more difficulty finding jobs than other workers displaced during non-recessionary periods. Then, it may be difficult to generalize the findings of this paper to the experience of displaced workers in other time periods.

## Appendix: Matching of the ATUS WB module and the DWS

Because the ATUS WB Module sample is drawn from the CPS, one can match observations from the January 2010 and 2012 DWS of the CPS to the 2010, 2012, and 2013 ATUS WB Modules by utilizing the rotation scheme in the CPS. The CPS is designed so that each household whose address is selected for the sample is repeatedly interviewed following a 4–8–4 sample rotation scheme: each month there are eight rotation groups in the CPS and a new rotation group of households enters the survey every month and is interviewed for four consecutive months, temporarily out for eight consecutive months, and then re-interviewed for four consecutive months before they are finally dropped from the CPS. Two to five months after the last CPS interview, some of these households are eligible for the ATUS interview. For example, a group of households that was in its 7th month of interview (Month in Sample, or MIS, 7) in the January 2010 DWS finished its 8th month of interview in the CPS in February 2010 and became eligible for the ATUS interview in April through July 2010. In the end, four rotation groups, those in MIS 5 through 8, from the January 2010 DWS and all eight rotation groups from the 2012 DWS can be matched to the 2010, 2012, and 2013 ATUS WB Modules. For these people, all ATUS interviews occurred between March and September. In the end, the time gap between the two surveys is 2 months at the minimum and 20 months at the maximum.

Following the guidelines from Bureau of Labor Statistics (2015), I first linked displaced workers and nondisplaced workers from the January 2010 and 2012 DWS to the 2010, 2012, and 2013 ATUS WB Modules by using a set of household and individual identification variables (HRHHID, HRHHID2, and PULINENO). Then I only kept the observations that had the same values for sex and race, and acceptable ranges of age difference between the two surveys (− 1 to 2 years for the 2010 and 2012 ATUS WB and − 1 to 3 years for the 2013 ATUS WB), as suggested by Madrian and Lefgren (2000). Column 3 of Table 8 shows that using a set of household and individual identification variables, among the sample of 84,323 individuals from the 2010 and 2012 DWS, 6413 individuals are successfully merged to the ATUS sample. Of these, 6360 observations remained after excluding cases with non-matching sex, race, and age between the two surveys. Among these individuals, the number of respondents who completed the ATUS WB Module interviews was 6051 respondents. Finally, Column 4 of Table 8 shows that the percentages of displaced workers remain more or less the same throughout this matching process, suggesting that displaced workers are not more likely to drop out of the surveys than nondisplaced workers.

**Table 8:** Number of Matched Individuals between the DWS and the ATUS WB Modules and the Final Sample Size

	(1) 2010 DWS MIS 5–8	(2) 2012 DWS MIS 1–8	(3) Total	(4) Displaced workers
DWS	28,744	55,579	84,323	7821 (9.28%)
Matched to ATUS by identification variables	2513	3900	6413	597 (9.31%)
Sex, race, and age verified	2508	3852	6360	590 (9.28%)
Complete ATUS WB Module interviews	2436	3615	6051	560 (9.25%)

In parentheses are the unweighted percentages of displaced workers out of the total sample

## Footnotes

1 This approach of using company closures as exogenous entry into unemployment to establish causality between unemployment and subjective well-being is now popular. For example, see Nikolova and Ayhan (forthcoming).

2 It is important to note that there are other explanations for the findings in Gibbons and Katz (1991). For example, using data from the National Longitudinal Survey of Youth, Krashinsky (2002) provides an alternative explanation that the reason why laid-off workers suffer larger wage losses is not because a layoff event reveals the lower ability of a worker, but rather because laid-off workers have more to lose from the beginning because they have been displaced from larger establishments with higher wages. On the other hand, Song (2007), based on the same DWS data used by Gibbons and Katz (1991), shows that the results in Gibbons and Katz (1991) partly stem from recall bias in the 1984 and 1986 DWS, but mostly reflect the fact that workers displaced by layoffs have significantly higher predisplacement wage-tenure profiles than do those displaced by plant closings, while there is no such difference in postdisplacement wage-tenure profiles.

3 Because job displacement is by definition involuntary entry into unemployment, the endogeneity problem raised by Kassenboehmer and Haisken-DeNew (2009) that a worker’s dissatisfaction may lead to voluntary entry into unemployment is not an issue here.

4 Other reasons include seasonal job completed, self-operated business failed, and some other reason. Such cases are counted as nondisplaced workers in the sample.

5 The selected activity must be at least 5 min in duration and the following activities and responses were not selected: sleeping, grooming, personal activities, don’t know/can’t remember, and refusal/none of your business. On average, about 15 activities per respondent were eligible for selection.

6 Because the question on the Cantril ladder was not included in the 2010 ATUS WB Module, the sample size is smaller for the Cantril ladder: 178 displaced workers and 1591 nondisplaced workers among men and 148 displaced workers and 1698 nondisplaced workers among women.

7 Beginning in the 2008 June CPS, the respondents were asked the following six questions on disability: (i) whether they have difficulty dressing or bathing; (ii) whether they are deaf or have serious difficulty hearing; (iii) whether they are blind or have serious difficulty seeing even when wearing glasses; (iv) whether they have difficulty doing errands alone such as visiting a doctor's office or shopping because of a physical, mental, or emotional condition; (v) whether they have serious difficulty walking or climbing stairs; and (vi) whether they have serious difficulty concentrating, remembering, or making decisions because of a physical, mental, or emotional condition. Since the ATUS sample is drawn from those who participated in the CPS, the responses to these questions are available for all ATUS WB respondents. Freedman et al. (2012) show that regardless of measures of subjective well-being, older married adults with disability reported worse subjective well-being than those without disability.

8 To account for the ordered nature of the responses to the Cantril ladder and self-rated health status, I also estimated Ordered Probit regressions and found the results are similar to the OLS regression results reported in this paper, consistent with the findings in Ferrer-i-Carbonell and Frijters (2004).

9 According to the CPS definition, family income is gross income before taxes and includes government transfers (https://www.census.gov/cps/data/incdef.html).

10 One may argue that because only three activities are selected per respondent for the ATUS WB Module, the measures of subjective well-being based on time use in columns 1 through 7 in Tables 2 and 3 might be subject to measurement error and that is why the coefficients on job displacement are insignificant for men in Table 2. However, the measurement error in the dependent variable only affects the standard errors. The fact that, based on almost the same sample size but with larger standard errors, many coefficients on job displacement are significant in Table 3 for women suggests that the different patterns by sex observed in Tables 2 and 3 are unlikely due to the measurement error in the dependent variable.

11 The missing dummy is for a small number of displaced workers, 3 men and 4 women, who failed to provide the exact year of displacement although they said they were displaced in the prior three years in the DWS.

## References

[CR1] Angner E (2010). Subjective well-being. J. SocioEcon..

[CR2] Black SE, Devereux PJ, Salvanes KG (2015). Losing heart? The effect of job displacement on health. Ind. Labor Relat. Rev..

[CR3] Brand JE, Levy BR, Gallo WT (2008). Effects of layoffs and plant closings on depression among older workers. Res. Aging.

[CR4] Brickman P, Campbell DT, Apley MH (1971). Hedonic relativism and planning the good society. Adaptation-level theory: a symposium.

[CR5] Browning M, Heinesen E (2012). Effect of job loss due to plant closing on mortality and hospitalization. J. Health Econ..

[CR6] Brülde B (2007). Happiness theories of the good life. J. Happiness Stud..

[CR7] Bungard SA, Brand JE, House JS (2007). Toward a better estimation of the effect of job loss on health. J. Health Soc. Behav..

[CR8] Cantril H (1965). The pattern of human concerns.

[CR9] Chaplin TM, Aldao A (2013). gender differences in emotion expression in children: a meta-analytic review. Psychol. Bull..

[CR10] Charles KK, Stephens M (2004). Job displacement, disability, and divorce. J. Labor Econ..

[CR11] Clark AE, Oswald AJ (1994). Unhappiness and unemployment. Econ. J..

[CR12] Clark AE, Georgellis Y, Sanfey P (2001). Scarring: the psychological impact of past unemployment. Economica.

[CR13] Doiron D, Mendolia S (2012). The impact of job loss on family dissolution. J. Popul. Econ..

[CR14] Eliason M (2012). Lost jobs, broken marriages. J. Popul. Econ..

[CR15] Eliason M, Storrie D (2009). Does job loss shorten life?. J. Hum. Resourc..

[CR16] Farber, H.S.: The incidence and costs of job loss: 1982–91. In: Brookings Papers on Economic Activity: Microeconomics 1993(1), pp. 73–119. (1993)

[CR17] Farber, H.S.: The changing face of job loss in the United States: 1981–1995. In: Brookings Papers on Economic Activity: Microeconomics 1997(1), pp. 55–128. (1997)

[CR18] Farber, H.S.: Job loss in the great recession: historical perspective from the displaced workers survey, 1984–2010. In: NBER Working Paper 17040. (2011)

[CR19] Ferrer-i-Carbonell A, Frijters P (2004). How important is methodology for the estimates of the determinants of happiness. Econ. J..

[CR20] Freedman VA, Stafford F, Schwarz N, Conrad F, Cornman JC (2012). Disability, participation, and subjective wellbeing among older couples. Soc. Sci. Med..

[CR21] Fujita F, Diener E, Sandvik E (1991). Gender differences in negative affect and well-being: the case for emotional intensity. J. Pers. Soc. Psychol..

[CR22] Gallo WT, Bradley EH, Siegel M, Kasl SV (2000). Health effects of involuntary job loss among older workers: findings from the health and retirement survey. J. Gerontol. Ser. B Psychol. Sci. Soc. Sci..

[CR23] Gallo WT, Bradley EH, Dubin JA, Jones RN, Falba TA, Teng H, Kasl SV (2006). The persistence of depressive symptoms in older workers who experience involuntary job loss: results from the health and retirement survey. J. Gerontol. Ser. B Psychol. Sci. Soc. Sci..

[CR24] Gallo WT, Teng H, Falba TA, Kasl SV, Krumholz HM, Bradley EH (2006). The impact of late career job loss on myocardial infarction and stroke: a 10 year follow up using the health and retirement survey. Occup. Environ. Med..

[CR25] Gelbach JB (2016). When do covariates matter? And which ones, and how much?. J. Labor Econ..

[CR26] Gerlach K, Stephen G (1996). A paper on unhappiness and unemployment in Germany. Econ. Lett..

[CR27] Gibbons R, Katz LF (1991). Layoffs and lemons. J. Labor Econ..

[CR28] Jacobson LS, LaLonde RJ, Sullivan DG (1993). Earnings losses of displaced workers. Am. Econ. Rev..

[CR29] Kahneman D, Krueger AB (2006). Developments in the measurement of subjective well-being. J. Econ. Perspect..

[CR30] Kahneman D, Krueger AB, Schkade DA, Schwarz N, Stone AA (2004). Toward national well-being accounts. Am. Econ. Rev..

[CR31] Kassenboehmer SC, Haisken-DeNew JP (2009). You’re Fired! The causal negative effect of entry unemployment on life satisfaction. Econ. J..

[CR32] Keefe V, Reid R, Ormsby C, Robson B, Purdie G, Baxter J, Ngäti Kahungunu Iwi Incorporated (2002). Serious health events following involuntary job loss in new zealand meat processing workers. Int. J. Epidemiol..

[CR33] Knabe A, Rätzel S, Schöb R, Weimann J (2010). Dissatisfied with life but having a good day: time-use and well-being of the unemployed. Econ. J..

[CR34] Kranshinsky H (2002). Evidence on adverse selection and establishment size in the labor market. Ind. Labor Relat. Rev..

[CR35] Krueger AB, Mueller AI (2012). Time use, emotional well-being, and unemployment: evidence from longitudinal data. Am. Econ. Rev..

[CR36] Krueger AB, Kahneman D, Schkade D, Schwarz N, Stone AA, Krueger AB (2009). National Time Accounting: The Currency of Life. Measuring the subjective well-being of nations: national accounts of time use and well-being.

[CR37] Madrian BC, Lefgren LJ (2000). An approach to longitudinally matching current population survey (CPS) respondents. J. Econ. Soc. Measure..

[CR38] Mestre MV, Samper P, Frías MD, Tur AM (2009). Are women more empathetic than men? a longitudinal study in adolescence. Spanish J. Psychol..

[CR39] Nikolova, M., Ayhan, S. H.: Your spouse is fired! How much do you care? J. Popul. Econ. (forthcoming)

[CR40] Noelke C, Beckfield J (2014). Recessions, job loss, and mortality among older US adults. Am. J. Public Health.

[CR41] Oswald AJ, Proto E, Sgroi D (2015). Happiness and productivity. J. Labor Econ..

[CR42] Schaller J, Stevens AH (2015). Short-run effects of job loss on health conditions, health insurance, and health care utilization. J. Health Econ..

[CR43] Song Y (2007). Recall bias in the displaced workers survey: are layoff really lemons?. Labour Econ..

[CR44] Stevens AH (1997). Persistent effects of job displacement: the importance of multiple job losses. J. Labor Econ..

[CR45] Strully KW (2009). Job loss and health in the U.S. labor market. Demography.

[CR46] Sullivan D, Wachter T (2009). Job displacement and mortality: an analysis using administrative data. Quart. J. Econ..

[CR47] U.S. Bureau of Labor Statistics. American time use survey user’s guide. (2015). http://www.bls.gov/tus/atususersguide.pdf. Accessed 28 July 2015

[CR48] Winkelmann L, Winkelmann R (1998). Why are the unemployed so unhappy? evidence from panel data. Economica.

[CR49] Wright TA, Cropanzano R (2000). Psychological well-being and job satisfaction as predictors of job performance. J. Occup. Health Psychol..

